# Two-Channel Portable Biopotential Recording System Can Detect REM Sleep Behavioral Disorder: Validation Study with a Comparison of Polysomnography

**DOI:** 10.1155/2022/1888682

**Published:** 2022-02-24

**Authors:** Hiroshi Kataoka, Tsunenori Takatani, Kazuma Sugie

**Affiliations:** Department of Neurology, Division of Central Clinical Laboratory, Nara Medical University, Kashihara, Nara, Japan

## Abstract

**Background:**

Sleep disorders are frequent nonmotor symptoms of Parkinson's disease (PD). Polysomnography (PSG) has been the gold standard for its assessment. However, it requires patients to stay overnight in a hospital or sleep center. The mobile two-channel electroencephalography (EEG)/electrooculography (EOG) recording system is a self-applicable and affordable method to objectively assess sleep at home. We aimed at evaluating patients with PD to confirm the difference in sleep parameters between the portable recording system and PSG.

**Methods:**

PSG and the portable recording system were simultaneously performed on a similar night in eight patients with PD. We compared the difference in sleep parameters between them using nonparametric tests.

**Results:**

All patients displayed a score of both PDSS −2 ≥ 15 and PSQI ≥ 5, respectively, which revealed poor sleep quality. There was no difference in the sleep parameters between the portable recording system and PSG, except for the percentage of sleep stage N3. Regarding the detection of REM sleep without atonia, we observed accordance between the portable recording system and PSG in six patients (*P*=0.686).

**Conclusions:**

The portable EEG/EOG recording system may gain an advantage from home-based evaluations for habitual sleep at home. Our study on device validation may contribute to measuring natural sleep, including rapid eye movement (REM) sleep behavioral disorder (RBD), in an outpatient care setting.

## 1. Introduction

In Parkinson's disease (PD), sleep disorders are frequent nonmotor symptoms with an estimated occurrence rate of 74–88% [1, 2], inducing sleep fragmentation and difficulties in maintaining sleep or falling asleep. Sleep fragmentation may increase the difficulties associated with PD pathology [3]. PD-related sleep disorders include nocturnal motor symptoms, psychosis, hallucinations, urinary incontinence, depression, cognitive impairment, vivid dreaming, and rapid eye movement (REM) sleep behavioral disorder (RBD). Since idiopathic RBD is the prodromal stage of PD [4] and PD with RBD has a more rapid progression with increased cognitive decline [5, 6], the importance of RBD has heightened.

Self-reported sleep diaries or questionnaires have been used extensively, with each method having its own strengths and limitations. To date, polysomnography (PSG) has been the gold standard assessment methodology to objectively measure the following conventional sleep parameters: sleep efficiency (SE), total sleep time (TST), wake time after sleep onset (WASO), and sleep onset latency (SOL). However, patients have to stay overnight in a hospital or sleep center for these tests. Thus, a self-applicable and affordable method is needed to measure sleep at home. Researchers have investigated several biomedical, mechanical, or kinetic devices for home-based sleep monitoring and have obtained some accuracy [7]. For example, the Mobile Health Systems Lab Sleep Band system consists of eight-channel biosignal electrodes on the headband and demonstrates the typical non-REM sleep pattern [8]. Wireless single-channel headband sleep systems reportedly have a moderate to high agreement with PSG in healthy participants [9]. Nonetheless, they cannot detect REM sleep without atonia (RWA), which is required for diagnosing REM behavior disorder (RBD). The mobile two-channel electroencephalography (EEG)/electrooculography (EOG) recording system (SleepGraph®, Proassist Co., Japan) is a self-applicable and affordable method to objectively assess sleep at home. The estimated sleep parameters were well correlated with those of PSG in a validation study of healthy adults [10, 11]. Moreover, this system can detect RWA and objectively diagnose RBD. We evaluated patients with PD to confirm the difference in sleep parameters between the portable EEG/EOG recording system and PSG. Our study on device validation may contribute to measuring natural sleep, including RBD, in an outpatient care setting.

## 2. Methods

We evaluated eight patients diagnosed with PD according to the International Parkinson and Movement Disorder Society (MDS) diagnostic criteria [12]. The clinical basic evaluations at the start of the study were the following: Hoehn–Yahr stage, MDS Revision of the Unified PD Rating Scale (UPDRS) parts 3 and 4 [13], subitem “psychosis” and “anxiety” on MDS-Non-Motor Rating Scale [14], Japanese version of the Montreal Cognitive Assessment (MoCA-J) [15], Japanese version of Parkinson's Disease Sleep Scale (PDSS)-2 [16], Japanese version of Sleep Behavior Disorder Screening Questionnaire (RBDSQ) [17], Pittsburgh Sleep Questionnaire Index (PSQI), and Beck depression score [18].

**Figure 1:**
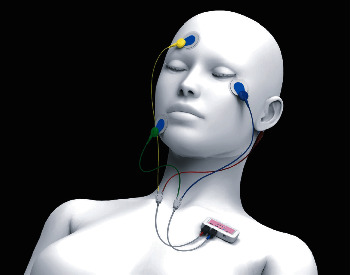
Portable recording system (ZA) (SleepGraph®, Proassist Co., Japan).

The portable recording system (SleepGraph®, Proassist Co., Japan; medical device certification number: 231AHBZX00001000) consisted of a pair of bipolar EEG and EOG electrode leads, and the receiver was used for frontal EEG and EOG recording (Figure 1) [10, 11]. The forehead EEG was recorded from Fp1 with the contralateral mastoid process (M2) as a reference. The EOG was recorded from two electrodes on the skin of opposing chin muscles approximately 1 cm below the eyes. The signal was recorded at a sampling frequency of 128 Hz using 0.540 and 0.544 EEG filters. Amplified and filtered analog data from the electrodes were converted into a digital signal using a 14-bit A/D converter, sent to a bedside-located receiver, and stored for offline data analysis. Sleep stage scoring was based on the forehead EEG signal [11], and in addition to the sleep stage structure, subsequent sleep measurements such as SE, TST, WASO, and SOL were calculated using the AASM rules [19]. When chin electromyography (EMG) activity, defined as the duration of phasic muscle activity lasting 0.1–5 seconds with an amplitude four times greater than that of the background, is occupied by more than 50% for mini epochs for 3 s, the epoch was defined as RWA. The ratio of RWA to total REM sleep (SREM) was calculated automatically.

### 2.1. Procedure

The PSG and the portable EEG/EOG recording system were simultaneously performed on the same night, and clinical evaluation was performed a few days prior. A standard PSG was performed and included six EEG signals (F3-M2, F4-M1, C3-M2, C4-M1, O1-M2, and O2-M1), two channels of EOG signals (E1-M2 and E2-M2), chin electromyography (EMG) (EMG1-EMG2 and EMG1-EMG3), and electrocardiography (ECG). The diagnostic PSG and portable EEG/EOG recording systems were manually scored by a certified sleep technologist with the definition of the AASM scoring manual [19]. The sleep technologist was not informed of any clinical information, except for age and gender. The study protocol was approved by the Medical Ethics Committee of the Nara Medical University.

### 2.2. Statistical Analysis

A comparison of the difference in sleep parameters between the portable EEG/EOG recording system and the PSG was performed using the nonparametric Mann–Whitney test and the Spearman correlation coefficient. The effect size was calculated using Cohen's *d*. Category was analyzed using the chi-square test. Statistical significance was set at *P* < 0.05. SPSS software (version 24) was used for the statistical analysis.

## 3. Results

The basic clinical characteristics are shown in Table 1. Three and five patients were in the early and late stages, respectively, and all patients had motor complications. Five patients had mild cognitive impairment defined by the MoCA-J, and nontroublesome visual hallucinations were observed in five patients. One patient received continuous intrajejunal infusion of levodopa-carbidopa intestinal gel (LCIG) (levodopa/carbidopa: 44/10.1 mg/h), and seven patients were administered long-acting dopamine agonists. All patients had a score of both PDSS-2 ≥15 and PSQI ≥5, which revealed poor sleep quality [20]. Six patients had mild depression, defined as 20 to 28 points on the BDI [21]. Five patients had a score of ≥5 on the RBDSQ [17].

**Figure 2:**
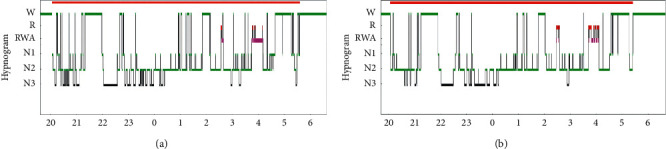
Hypnogram. (a) Polysomnography. (b) Portable recording system (ZA) (SleepGraph®, Proassist Co., Japan).

No difference was observed in sleep parameters between the portable EEG/EOG recording system and PSG, except for sleep stage N3 (Table 2 and Figure 2). Sleep efficiency, WASO, SOL, sleep period time (SPT), percentage of REM periods, and RWA/SREM on the portable EEG/EOG recording system were significantly correlated with PSG. As for the detection of RWA (Table 3), the accordance between the portable EEG/EOG recording system and PSG was found in six out of eight patients (*P*=0.686).

## 4. Discussion

The present study showed that TIB, SPT, WASO, SE, and REM period percentages were not significantly different between the portable EEG/EOG recording system and PSG. Also, a significant correlation was found between these sleep parameters, similar to previous validation studies of healthy subjects [10, 11]. The TST and sleep stage N1 and N2 results did not show a significant correlation with the Spearman correlation coefficient, and there was a significant difference for stage N3 using the Mann–Whitney test. From the validation study of the portable EEG/EOG recording system, a correlation for stages N1, N2, and N3 between two independent scorers was not consistent on Bland–Altman plots, and a dissociation of the *P* value between the two scorers was observed for these stages [11]. K complex and delta waves that characterize stages N2 and N3 can be detected from the electrodes located in the frontal lobe region [22, 23], but alpha and sleep spindle waves are more clearly observable in the central and occipital EEG [22–25]. Thus, the forehead EEG electrodes have less power to detect certain characteristic EEG waves which identify sleep stages.

The importance of RBD has been increasing from the aspect of the prodromal stage of neurodegenerative diseases such as PD and the prediction of disease progression [5]. For example, PD patients with RBD have a higher incidence of dementia or worse autonomic dysfunction [6, 26]. A strength of the present study is that the portable EEG/EOG recording system can detect RWA, although the percentage of RWA seems to be slightly different between the portable EEG/EOG recording system and PSG. The diagnosis of RBD is recognized worldwide and requires the detection of RWA on PSG. However, PSG is labor-intensive, time-consuming, and expensive [27], and the subject had to stay overnight in a laboratory setting. Due to the highly consistent detection of RWA on the portable EEG/EOG recording system and PSG, it is expected that RWA in addition to natural sleep can be evaluated in outpatients. A discordance of RWA detection between the portable EEG/EOG recording system and PSG was observed in two patients, but they did not have a score ≥5 on the RBDSQ. The limitations of the present study are the small sample size and the recording of results being undertaken by a single expert sleep scorer. Fewer electrodes in the portable recording system than that in PSG may have led to an error in diagnosis, such as phasic-activity type RWA.

In conclusion, sleep parameters between the portable EEG/EOG recording system and PSG are likely to be similar rather than sleep stages N1, N2, and N3, and this may gain an advantage from home-based evaluations for habitual sleep at home, particularly in subjects who are difficult to evaluate otherwise. The two-channel portable EEG/EOG recording system may be a suitable technique for diagnosing RBD, especially in an outpatient care setting.

## Figures and Tables

**Table 1:** Basic characteristics of subjects with Parkinson's disease.

	Patient 1	Patient 2	Patient 3	Patient 4	Patient 5	Patient 6	Patient 7	Patient 8
Age/gender	67/M	57/M	66/F	70/F	61/M	50/M	78/M	76/F
Disease duration (years)	5	11	11	12	11	10	10	17
Hypertension	−	−	−	**+**	−	−	−	−
Body mass index	20.5	19.4	20.2	22.6	27.2	19.4	21.7	16.1
Hoehn–Yahr stage	2	2	2	4	4	4	4	5
MDS-UPDRS part 3	32	10	7	38	22	25	28	57
MDS-UPDRS part 4	5	11	4	7	7	7	4	7
MoCA-J	25	23	26	22	25	29	26	23
Psychosis^*∗*^	0	2	2	11	4	0	3	0
Anxiety^*∗*^	2	12	0	3	17	6	6	2
Levodopa (mg/day)	50	700	600	400	550	LCIG	1100	400
Dopamine agonists (DA)	**+**	**+**	**+**	**+**	**+**	−	**+**	**+**
Long-acting DA	**+**	**+**	**+**	**+**	**+**	−	**+**	**+**
Sleeping drug	−	**+**	−	**+**	**+**	−	−	**+**
PDSS-2 total score	17	32	21	21	20	27	43	36
Disturbed sleep on PDSS-2	10	15	14	13	9	14	15	10
Motor symptoms at night on PDSS-2	4	9	4	2	5	8	15	11
PD symptoms at night on PDSS-2	3	7	3	6	6	5	13	14
PSQI total score	6	14	13	15	14	10	15	10
Beck depression score	14	28	16	21	23	20	27	20
RBD score	9	7	2	9	2	4	12	6

MDS-UPDRS: Movement Disorder Society Revision of the Unified PD Rating Scale; LCIG: continuous intrajejunal infusion of levodopa-carbidopa intestinal; MoCA-J: Japanese version of the Montreal Cognitive Assessment; PDSS: PD Sleep Scale; PSQI: Pittsburgh Sleep Questionnaire Index; RBD: REM sleep behavior disorder. ^*∗*^MDS-Non-Motor Rating Scale.

**Table 2:** Difference of sleep parameters between polysomnography and the portable EEG/EOG recording system.

	PSG		Portable EEG/EOG recording system		Mann–Whitney	Effect size	Spearman rank correlation	
	Mean	SD	Mean	SD	*P*	Cohen d	*P*	Correlation coefficient
TST (min)	381.8	58.9	394.1	103.9	0.753	0.15	0.693	0.167
TIB (min)	644.1	56.8	673	65.6	0.371	0.47	0.568	0.24
SPT (min)	507.2	95.8	508	92.8	1	0.01	0.01^†^	0.833
SOL (min)	89	81.3	94.7	77.2	0.793	0.07	0.001^†^	0.929
WASO (times)	30.7	9.7	25.5	12.3	0.246	0.47	0.003^†^	0.896
WASO (min)	117.8	50.1	136	69.4	0.6	0.3	0.058	0.69
WASO (%)	29.9	10.3	39.8	26.4	0.674	0.49	0.002^†^	0.905
SE (%)	60.6	7.8	55.5	11.7	0.345	0.51	0.015^†^	0.81
REM (%)	6.7	7.7	13.3	10.9	0.093	0.70	<0.001^†^	0.976
N1 (%)	10.4	3.7	8.8	2.9	0.401	0.48	0.086	0.643
N2 (%)	52.7	6.4	55.9	10.5	0.401	0.37	0.12	0.595
N3 (%)	25	6.3	19.5	4.3	0.036^†^	1.02	0.289	0.429
RWA (%)	5	6	1.9	1.8	0.293	0.70	0.435	0.323
RWA/total SREM (%)	37.3	36.1	17.1	13.6	0.431	0.74	0.028^†^	0,762

TST: total sleep time; TIB: time in bed; SPT: sleep period time; WASO: wake time after sleep onset; SOL: sleep onset latency; SE: sleep efficiency; EEG/EOG: electroencephalography/electrooculography, †: *P* < 0.05.

**Table 3:** Detection of REM sleep without atonia (RWA).

	Patient 1	Patient 2	Patient 3	Patient 4	Patient 5	Patient 6	Patient 7	Patient 8
Polysomnography	**+**	**+**	**+**	**+**	−	**+**	**+**	**+**
Portable EEG/EOG recording system	**+**	**+**	**+**	**+**	**+**	−	**+**	**+**
RBD score ≥5	**+**	**+**	−	**+**	−	−	**+**	**+**

RBD: rapid eye movement (REM) sleep behavioral disorders.

## Data Availability

The data used to support the findings of this study are available from the corresponding author upon request.
